# CXCL17 Is Dispensable during Hypervirulent *Mycobacterium tuberculosis* HN878 Infection in Mice

**DOI:** 10.4049/immunohorizons.2100048

**Published:** 2021-09-24

**Authors:** José Alberto Choreño-Parra, Micah D. Dunlap, Rosemary Swanson, Luis A. Jiménez-Álvarez, Marcela Muñoz-Torrico, Silvia Guzmán-Beltrán, Joaquín Zúñiga, Shabaana A. Khader

**Affiliations:** * Instituto Nacional de Enfermedades Respiratorias Ismael Cosío Villegas, Mexico City, Mexico;; † Escuela Nacional de Ciencias Bilógicas, Instituto Politécnico Nacional, Mexico City, Mexico;; ‡ Department of Molecular Microbiology, Washington University School of Medicine, St. Louis, MO;; § Clinica de Tuberculosis, Instituto Nacional de Enfermedades Respiratorias Ismael Cosío Villegas, Mexico City, Mexico;; ¶ Investigación en Microbiología, Instituto Nacional de Enfermedades Respiratorias Ismael Cosío Villegas, Mexico City, Mexico;; ‖ Tecnologico de Monterrey, Escuela de Medicina y Ciencias Biomédicas, Mexico City, Mexico

## Abstract

CXCL17 is a novel mucosal chemokine that mediates myeloid cell recruitment and bactericidal activity and highly expressed in the respiratory tract. However, its role in tuberculosis (TB) immunopathogenesis or protection remains unknown. In this study, we evaluated the function of CXCL17 in a mouse model of aerosol infection with the clinical W-Beijing lineage *Mycobacterium tuberculosis* hypervirulent HN878 strain. Our results show that CXCL17 production increases in the lung of *M. tuberculosis*–infected mice during acute and chronic stages of infection. Moreover, in vitro *M. tuberculosis* infection of epithelial cells and myeloid cells induces production of CXCL17. In humans, lower serum CXCL17 levels are observed among active pulmonary TB patients when compared with subjects with latent TB infection and healthy controls, suggesting a protective role. However, mice treated with rCXCL17 show similar lung bacterial burden and inflammation compared with control animals, despite an increased lung myeloid cell accumulation. Finally, CXCL17^−/−^ mice are not more susceptible to TB than wild-type animals. These findings suggest that CXCL17 is induced in both murine epithelial and myeloid cells upon *M. tuberculosis* infection and increased expression during human latent TB infection. However, CXCL17 may have a dispensable role during pulmonary TB.

## INTRODUCTION

*Mycobacterium tuberculosis*, the causative agent of tuberculosis (TB), is a leading infectious cause of death worldwide. Host immunity against *M. tuberculosis* requires a balanced interaction of different chemokine/chemokine receptor axes controlling leukocyte recruitment into the lung (1). Blocking pathological chemokine-mediated activities or harnessing beneficial chemokine/receptor axes would constitute critical therapeutic strategies for TB. This emphasizes the importance of a complete understanding of the role of chemokines during TB, especially of those that are yet to be assigned a specific function.

CXCL17 is a novel mucosal chemotactic factor for myeloid cells that also possesses a broad spectrum of bactericidal activity (2–4). This chemokine is expressed along the respiratory epithelium at a steady state and under inflammatory conditions (2, 3, 5), making it a potential participant in mediating lung defense against pathogens. CXCL17 induces rigorous migration of monocytes and dendritic cells (DCs) via ERK1/2 and p38 MAPK signaling (2, 6). These chemotactic properties might be of special relevance during TB because the myeloid cell infiltration into the lung is a crucial determinant of the protective versus pathogenic nature of tubercle granulomas. For instance, the presence of CCR2^+^ alveolar macrophages (AMs) in the airway is pivotal for the initiation of anti–*M. tuberculosis* immunity. Upon engulfing bacilli, these cells migrate to the lung parenchyma and promote the formation of protective granulomas (7). Conversely, interstitial IFN-responsive macrophages and plasmocytoid DCs might be more permissive for *M. tuberculosis* growth or induce inflammatory reactions that result in lung tissue damage during active TB (8).

Interestingly, healthy CXCL17^−/−^ mice have decreased numbers of AMs but an intact number of interstitial lung macrophages at the steady state (4). Therefore, CXCL17 may also participate in the early regulation of the myeloid cell composition of TB granulomas. We previously showed overexpression of the *CXCL17* gene among innate lymphoid cells isolated from the lung of TB patients (9). However, the role of CXCL17 in protective immunity against *M. tuberculosis* remains elusive. In the current study, we addressed whether CXCL17 participates in immune protection against TB in a model of hypervirulent *M. tuberculosis* HN878 infection in mice. Our results indicate that murine *M. tuberculosis* HN878 infection induces CXCL17 production within the lung and in both epithelial and myeloid cells after early and late time points following infection. Furthermore, CXCL17 treatment attracts monocytes to the site of *M. tuberculosis* HN878 infection. In *M. tuberculosis*–infected individuals, serum levels of CXCL17 are higher among latent TB-infected (LTBI) subjects than individuals with active pulmonary TB (PTB), suggesting a protective role. However, CXCL17^−/−^ mice are not more susceptible to *M. tuberculosis* HN878 infection than wild-type (WT) controls. These findings suggest that CXCL17 is induced in murine epithelial and myeloid cells upon *M. tuberculosis* exposure, and increased levels accumulate in human LTBI. However, CXCL17 expression is dispensable following hypervirulent *M. tuberculosis* HN878 infection in mice.

## MATERIALS AND METHODS

### Human samples

Peripheral blood samples were obtained from individuals with active PTB and LTBI that were recruited at the TB clinic of the Instituto Nacional de Enfermedades Respiratorias Ismael Cosío Villegas in Mexico City, Mexico. Subjects with immuno-deficiencies, patients with diabetes, and those taking anti-TB drugs before recruitment were not eligible. A group of healthy individuals was also included and served as healthy controls (HC). Clinical and demographic data from participants were obtained by direct clinical interview, physical examination, and review of their medical records. PTB patients were evaluated by chest x-ray images, and the degree of lung damage was determined using a quantitative scale, as previously described (10, 11). Briefly, lung parenchyma was divided in four quadrants, with the division between the bilateral upper and lower lung set at the carina section. Each quadrant was scored from 0–5, where 0 denoted absence of lesions and 5 denoted extensive effect. All patients provided written informed consent to participate in the study. The Institutional Review Board of the Instituto Nacional de Enfermedades Respiratorias Ismael Cosío Villegas approved the study.

### Mice

WT C57BL/6 (B6) mice were purchased from The Jackson Laboratory (Bar Harbor, ME). Cxcl17^tm1b^ knockout mice (CXCL17^−/−^) were obtained from the Knockout Mouse Repository (University of California Davis, Davis, CA). Animals were bred at the animal facility in the Washington University School of Medicine (St Louis, MO). Experimental mice were age and sex matched and used between the ages of 6–8 wk, in accordance with the Institutional Animal Care and Use Committee guidelines at Washington University, and approved under protocol 20160129.

### *M. tuberculosis* infection and CXCL17 treatment

*M. tuberculosis* HN878 (BEI Resources, Manassas, VA; National Institutes of Health contract AI-75320) and *M. tuberculosis* H37Rv were cultured in Proskauer–Beck medium containing 0.05% Tween 80 to reach midlog phase and frozen in 1-ml aliquots at −80°C until used. Mice were aerosol-infected with ~100 CFUs of *M. tuberculosis* HN878 using a Glas-Col airborne infection system and euthanized at given time points. Some B6 mice received ~100 mg/kg mouse rCXCL17 (4270-DM/CF; R&D Systems, Minneapolis, MN) administered intratracheally in 50 μl of sterile PBS per mouse at different days post–*M. tuberculosis* HN878 infection (dpi). Pulmonary bacterial burden was determined by plating 10-fold serial dilutions of lung homogenates on Petri dishes containing 7H11 agar solid medium and counting CFUs.

### In vitro infections

Total lung cell suspensions, C10 epithelial cells, bone marrow–derived macrophages (BMDMs), and bone marrow–derived DCs (BMDCs) were obtained and cultured as described before (7). Cells were infected with *M. tuberculosis* HN878 at a multiplicity of infection of 1 in antibiotic-free complete DMEM, and culture supernatants were collected after 48 h.

### Histology

Mouse lungs were inflated with 10% neutral buffered formalin and embedded in paraffin. Five-micrometer lung sections were stained with H&E and processed for light microscopy. For calculation area of inflamed lung sections, representative images were taken with the Hamamatsu NanoZoomer 2.0-HT system with NDP scan image acquisition software. The percentage area occupied by inflammation was calculated as described before (12).

### Flow cytometry

At given dpi, lung single-cell suspensions were obtained and treated with Fc Block (CD16/CD32,2.4G2; Tonbo Biosciences, San Diego, CA) as previously described (7). Lung cells were incubated with fluorochrome-labeled specific Abs: CD11c (HL3; BD Biosciences), CD11b (M1/70; BD Biosciences and Tonbo Biosciences), SiglecF (E50–2440; BD Biosciences), and Gr-1 (RB6–8C5; BD Biosciences). Cells were acquired using a BD LSR Fortessa flow cytometer and gated based on their forward and side scatter characteristics. Neutrophils, monocytes, recruited macrophages (RMs), myeloid DCs (mDCs), and AMs were defined as before (7). The frequency of specific cell types was calculated using FlowJo (Flow Jo, Ashland, OR).

### CXCL17 quantification

Mouse and human CXCL17 levels in lung homogenates, culture supernatants, and serum samples were determined by ELISA using commercial kits (MBS916471 and MBS7232792; MyBio-Source, San Diego, CA).

### Lung cytokine expression

Total lung RNA was extracted using the RNeasy Mini Kit (QIAGEN), and DNase I (QIAGEN)–treated cDNA was generated using Applied Biosystems (ABI) reverse transcription reagents (ABI/Thermo Fisher Scientific) and then amplified with FAM-tagged probes and real-time PCR primers on a ViiA7 Real-Time PCR System (Life Technologies/Thermo Fisher Scientific). Specific gene expression was calculated relative to *GAPDH* mRNA expression. Primer and probe sequences targeting specific genes (IL-1β, TNF-α, IFN-γ, IL-17, and IL-10) were commercially purchased (ABI).

### Bactericidal assay

A stock of *M. tuberculosis* HN878 and H37Rv was thawed, and 500 μl was transferred to a sterile glass tube containing Proskauer–Beck medium. The cultures were incubated at 37°C, 7.5% CO_2_, with continuous shaking (100 rpm) for 7 d to allow bacteria to reach midlog phase. Afterward, 1ml from each culture was taken and diluted (1:75 for *M. tuberculosis* HN878, 1:50 for *M. tuberculosis* H37Rv) in Proskauer–Beck medium. The bacterial suspension was placed in a 96-well plate by adding 100 μl/well. An extra 100 μl of human rCXCL17 (0.5μM, 1μM, and 2μM) was added to the corresponding wells (final volume of reaction 200 μl/well) using saline solution and rifampicin (5μg/ml) as negative and positive controls, respectively. Treated bacteria were incubated at 37°C, 7.5% CO_2_, for 5 d, then 10-fold serial dilutions of the cultures were plated on Petri dishes containing 7H11 agar solid medium, and CFUs were determined after 14–21 d. Each condition was performed in triplicate.

### Statistical analysis

Statistical analyses were performed using GraphPad Prism 5 (GraphPad, La Jolla, CA). Specific analysis tests are mentioned in the figure legends. The *p* values ≤ 0.05 were considered significant (*p ≤ 0.05, **p ≤ 0.01, ***p ≤ 0.001, and ****p ≤ 0.0001).

## RESULTS

### *M. tuberculosis* HN878 infection-induced CXCL17 production in mouse lungs

**FIGURE 1.**
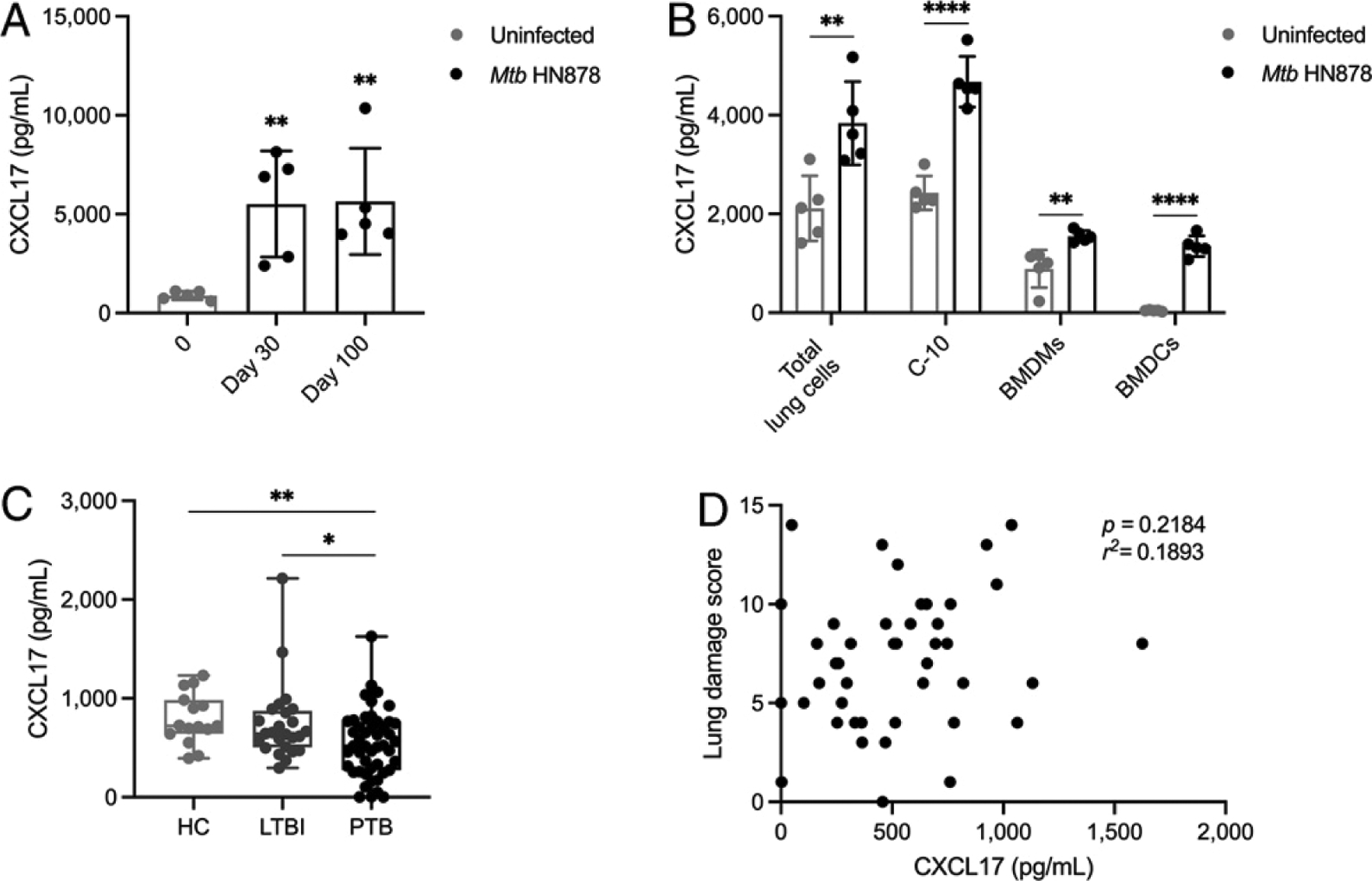
Induction of CXCL17 in mice and humans with TB. (**A**) B6 mice were infected with ~100 CFU of aerosolized *M. tuberculosis* HN878, and CXCL17 levels were measured at 30 and 100 dpi in lung homogenates. The CXCL17 levels in *M. tuberculosis*–infected mice were compared with basal levels of chemokine production in lung homogenates from uninfected animals (*n* = 5 mice per time point) using the Kruskal–Wallis test and the post hoc Dunn test for multiple comparisons. (**B**) Total lung cell suspensions, C-10 mouse lung epithelial cells, BMDMs, and BMDCs, were infected with *M. tuberculosis* HN878 at a multiplicity of infection =1 for 48 h. Levels of CXCL17 in supernatants from infected and uninfected cells were compared using the unpaired Student *t* test (*n* = 5 per group). The data shown represent mean (± SD) values from two to three independent experiments per time point and experimental condition. (**C**) Levels of CXCL17 in serum from HC (*n* = 16), subjects with LTBI (*n* = 25), and patients with active PTB (*n* = 47) were compared using the Kruskal–Wallis test and the post hoc Dunn multiple comparisons test. (**D**) The correlation between serum levels of CXCL17 and values obtained in a radiological lung damage score in PTB patients was analyzed using the Pearson correlation coefficient (*n* = 47). *p ≤ 0.05, **p ≤ 0.01, ****p ≤ 0.0001.

Different microbial stimuli are known to induce CXCL17 expression (4, 6, 13). To address whether CXCL17 is induced during *M. tuberculosis* infection, B6 mice were infected with the clinical W-Beijing lineage *M. tuberculosis* hypervirulent strain HN878, and CXCL17 levels were determined in lung homogenates. We observed increased CXCL17 production in the lung of infected animals at both acute and chronic stages of TB when compared with levels measured in uninfected animals (Fig. 1A). Using total lung cell suspensions from B6 mice, we also found that the in vitro exposure to *M. tuberculosis* HN878 induced increased CXCL17 production in lung cells (Fig. 1B). To further evaluate possible cellular sources of this chemokine during TB, mouse lung epithelial cells, BMDMs, and BMDCs were exposed to *M. tuberculosis* HN878 in vitro. Indeed, all cell subtypes produced high amounts of CXCL17 after the bacterial exposure (Fig. 1B), suggesting that both epithelial and myeloid cellular sources produce CXCL17 in response to *M. tuberculosis* infection.

To address whether CXCL17 was induced in individuals infected with *M. tuberculosis*, we measured CXCL17 levels in serum collected from LTBI and PTB patients. We did not observe an increase in serum levels of CXCL17 in humans with either LTBI or PTB compared with HC (Fig. 1C). Indeed, PTB patients showed a significant decrease in serum CXCL17 amounts when compared with LTBI and HC, whereas no differences between HC and LTBI were observed. Additionally, CXCL17 levels did not correlate with increased lung damage as measured by a validated radiological score among PTB patients (Fig. 1D). These data suggest that, whereas CXCL17 expression is induced in lungs during TB in mice, expression in the peripheral blood is decreased in individuals with PTB.

### CXCL17 expression is dispensable for host immunity in the mouse model

**FIGURE 2.**
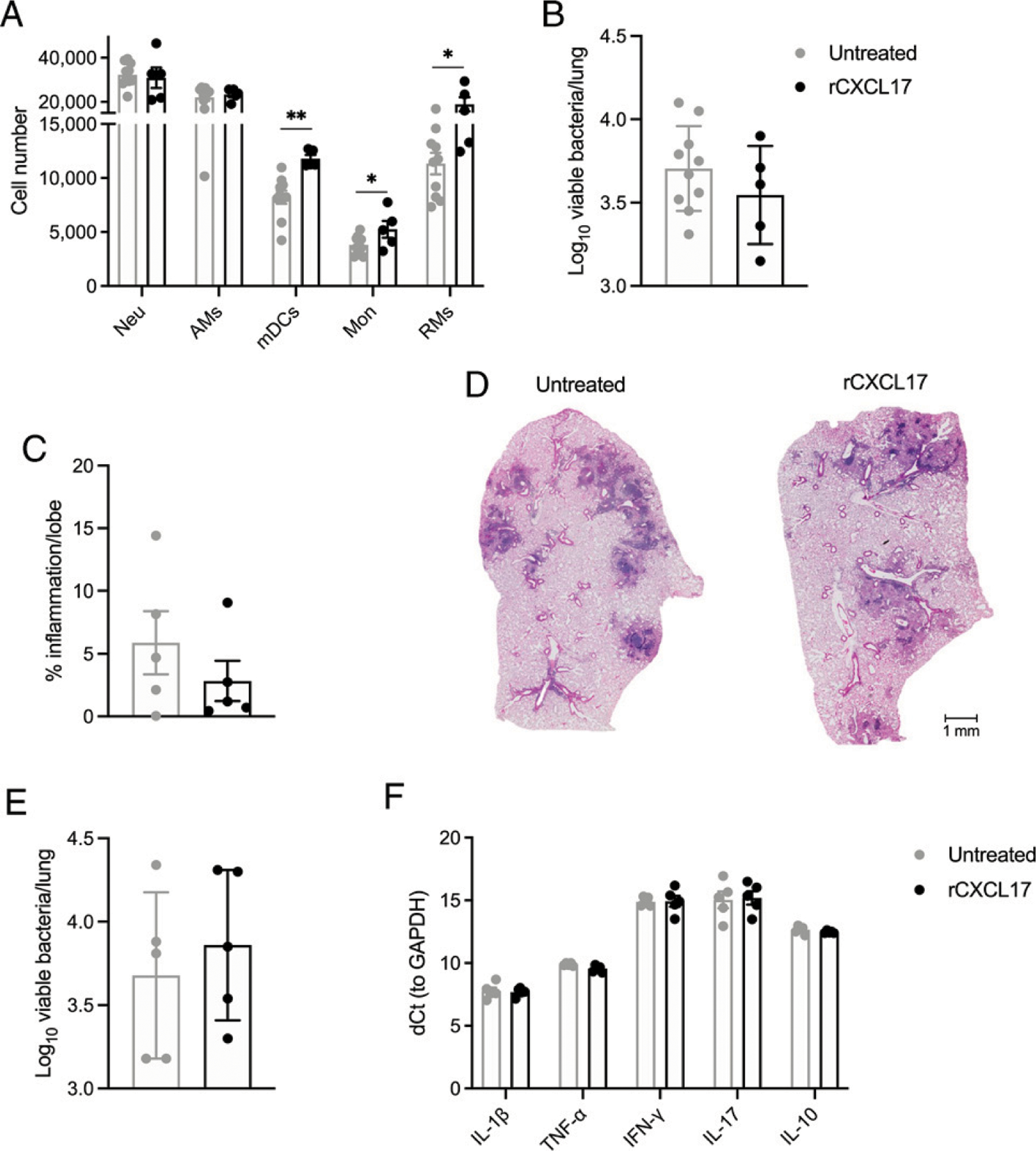
Administration of rCXCL17 does not improve the control of *M. tuberculosis* HN878 infection in mice. B6 mice were infected with ~100 CFU of aerosolized *M. tuberculosis* HN878 as before and sacrificed at 30 dpi. Some mice (*n* = 5) received ~100mg/kg rCXCL17 administered by the intratracheal route at 0, 7, and 14 dpi, whereas a separate group of animals (*n* = 10) were treated with PBS. (**A**) Lung myeloid cell populations were enumerated in *M. tuberculosis* HN878-infected B6 mice treated with rCXCL17 and control animals using flow cytometry. (**B**) Lung bacterial burden was determined by plating and counting CFUs. (**C**) Lung area occupied by inflammation was quantified in H&E-stained, formalin-fixed, and paraffin-embedded (FFPE) lungs using NanoZoomer software. (**D**) Representative microphotographs showing inflammation in H&E-stained FFPE lung sections (original magnification ×10) from rCXCL17-treated and untreated *M. tuberculosis* HN878-infected B6 mice. (**E**) Another group of B6 mice infected with *M. tuberculosis* HN878 was treated with rCXCL17 and sacrificed at 16 dpi. Lung bacterial burden was determined by plating. (**F**) The expression of several cytokines in the lungs of mice was assessed by real-time PCR. Comparisons between groups were performed using the unpaired Student *t* test. The data shown represent mean (± SD) values from two to three independent experiments. *p ≤ 0.05, **p ≤ 0.01.

To better characterize a functional role for CXCL17, we used the mouse model of hypervirulent *M. tuberculosis* infection. We first tested the effect of the exogenous rCXCL17 treatment on *M. tuberculosis* HN878 infection. Two groups of B6 mice were infected with *M. tuberculosis* HN878; one group was treated with rCXCL17 at 0, 7, and 14 dpi, and the other received PBS. At 30 dpi, we measured immune cell recruitment and found that *M. tuberculosis* HN878-infected mice treated with rCXCL17 showed increased recruitment of mDCs, monocytes, and RMs in the infected lung when compared with PBS-receiving control *M. tuberculosis* HN878-infected lungs (Fig. 2A). No differences in the number of neutrophils and AMs were observed between animal groups (Fig. 2A). In contrast, lung bacterial burden and lung inflammation measured was similar between CXCL17-treated and control *M. tuberculosis* HN878-infected lungs (Fig. 2B–D).

Given the possibility that the dosage, half-life, and timing of rCXCL17 administration was inadequate to observe a strong effect on *M. tuberculosis* CFU at 30 dpi, we then analyzed another group of mice infected with *M. tuberculosis* HN878 and treated with rCXCL17 but sacrificed 48 h after the last dose (16 dpi). Again, no differences in lung bacterial burden were observed as compared with mice that received PBS (Fig. 2E). Additionally, rCXCL17 treatment did not impact expression of cytokines in total lung tissue, as we did not find any differences in the expression of IL-1β, TNF-α, IFN-γ, IL-17, and IL-10 between *M. tuberculosis* HN878-infected B6 mice receiving rCXCL17 when compared with *M. tuberculosis*–infected mice treated with PBS (Fig. 2F). Together, these data demonstrate that increased signaling through the CXCL17/CXCL17–receptor axis improved myeloid cell recruitment to the lungs of B6 mice without improving overall *M. tuberculosis* control and lung tissue inflammation.

**FIGURE 3.**
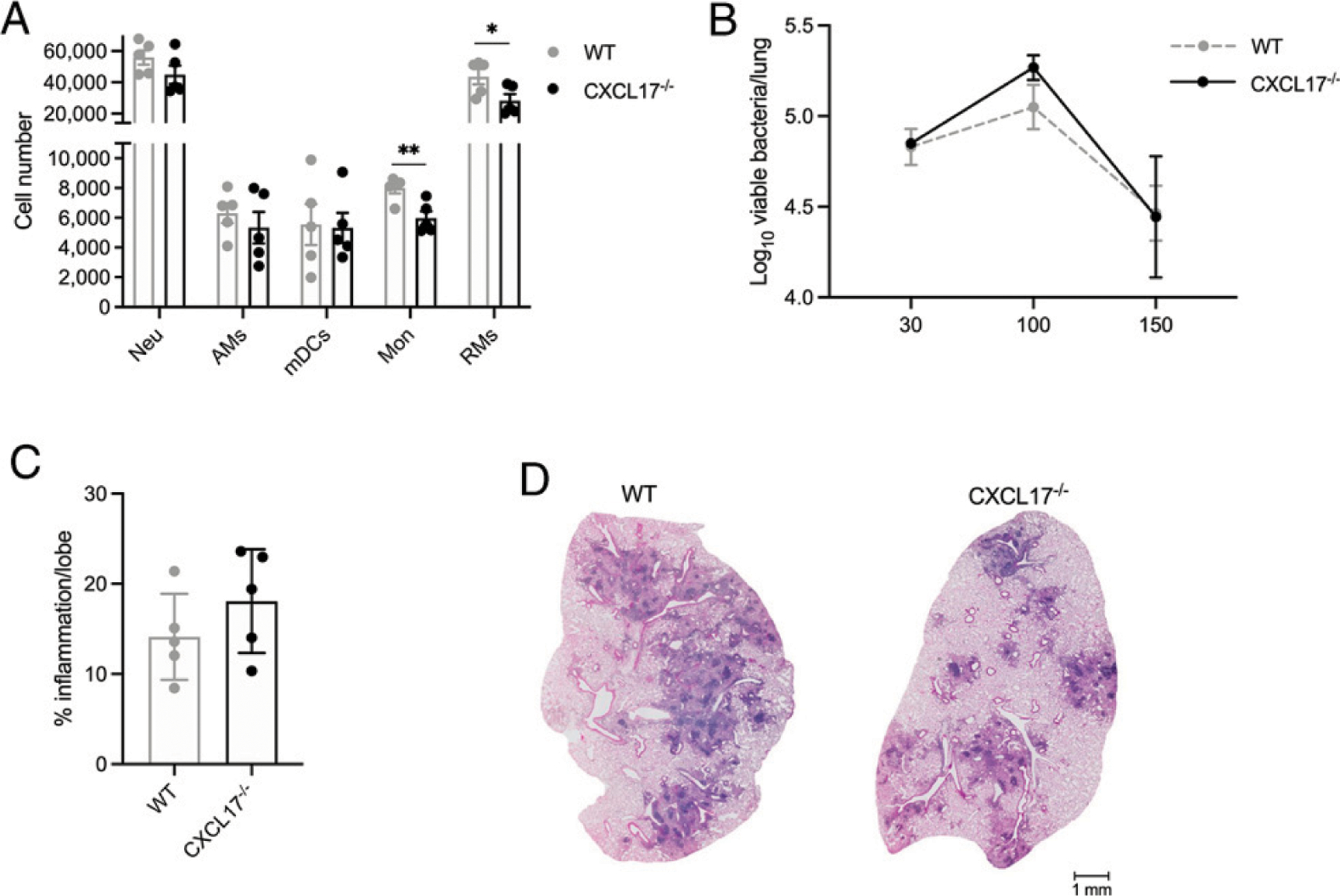
*M. tuberculosis* HN878 infection in CXCL17^−/−^ mice. WT B6 and CXCL17^−**/**−^ mice were aerosol-infected with ~100 CFU of *M. tuberculosis* HN878 and sacrificed at given dpi (*n* = 5 per group per time point). (**A**) Lung myeloid cell populations were enumerated in *M. tuberculosis* HN878-infected WT and CXCL17^−/−^ mice using flow cytometry at 30 dpi. (**B**) Lung bacterial burden was determined by plating and counting CFUs at 30, 100, and 150 dpi. (**C**) Lung area occupied by inflammation was quantified in H&E-stained, formalin-fixed, and paraffin-embedded (FFPE) lungs using NanoZoomer software. The percentage of lung area occupied by inflammation per mouse lung was determined at 100 dpi. (**D**) Representative microphotographs showing H&E-stained FFPE lung sections from *M. tuberculosis* HN878-infected WT and CXCL17^−/−^ mice at 100 dpi. Comparisons between groups were performed using the unpaired Student *t* test at each time point. The data shown represent mean (± SD) values from two to three independent experiments. *p ≤ 0.05, **p ≤ 0.01.

Finally, we evaluated immune protection against TB in the absence of CXCL17 signaling. WT and CXCL17^−/−^ mice were infected with *M. tuberculosis* HN878 as before. After 30 dpi, CXCL17^−/−^ showed impaired recruitment of monocytes and RMs to the lung, with no differences in the number of mDCs, AMs, and neutrophils with respect to WT mice (Fig. 3A). However, CXCL17^−/−^ mice showed similar levels of lung bacterial burden at 30, 100, and 150 dpi compared with WT controls (Fig. 3B). Furthermore, no differences in lung inflammation occurred between animal groups at 100 dpi (Fig. 3C, 3D). Hence, our data indicate that, whereas CXCL17 is induced in the murine lung during PTB, CXCL17 expression is not required for protective immunity against *M. tuberculosis* HN878 infection in mice.

### CXCL17 lacks bactericidal activity against *M. tuberculosis*

**FIGURE 4.**
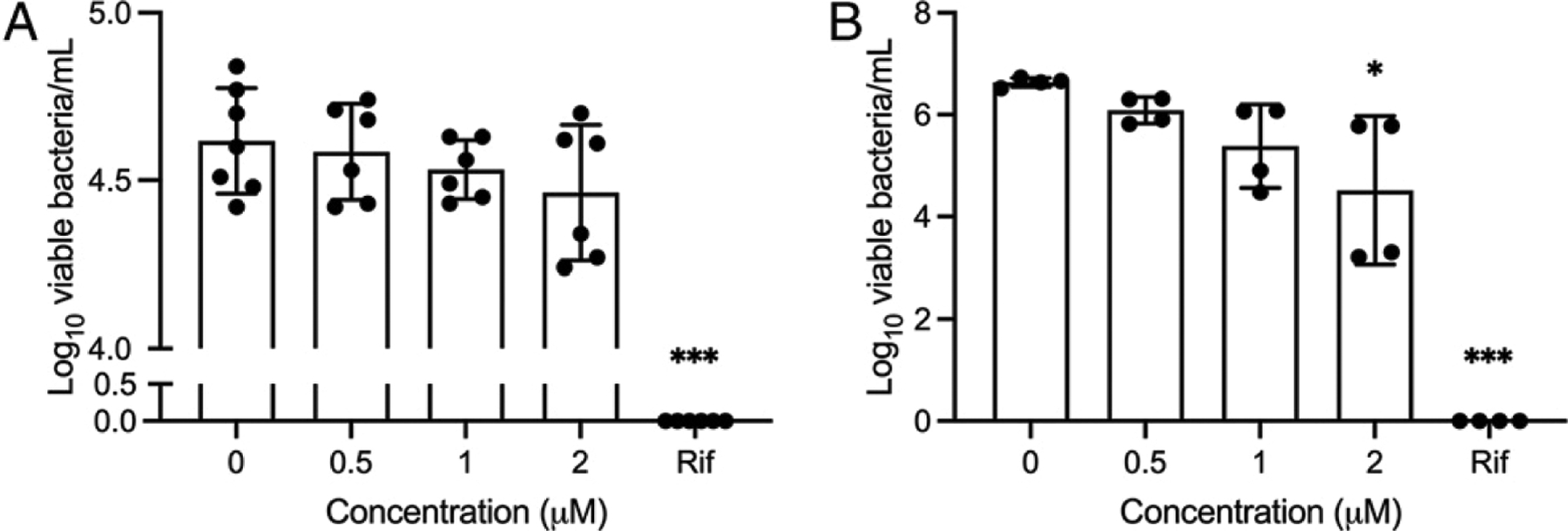
Bactericidal activity of CXCL17 against *M. tuberculosis*. Exponentially growing *M. tuberculosis* HN878 (**A**) and *M. tuberculosis* H37Rv (**B**) were treated with human rCXCL17 (see *Materials and Methods*). After chemokine exposure, the bacteria were spread on 7H11 agar solid medium plates and incubated at 37°C for 14–21 d. Surviving bacteria were counted as CFU/ml at each concentration. Saline solution and rifampicin (Rif) were used as negative and positive controls, respectively. Data were analyzed using the Kruskal–Wallis test and the post hoc Dunn multiple comparisons test. *p ≤ 0.05, ***p ≤ 0.001.

An important property of CXCL17 is that this chemokine can disrupt the membrane of *Escherichia coli*, *Staphylococcus aureus*, *Salmonella sp*., *Pseudomonas aeruginosa*, and *Candida albicans* (3). To address a lytic effect on *M. tuberculosis*, we cultured *M. tuberculosis* HN878, and the laboratory adapted *M. tuberculosis* strain H37Rv in presence of CXCL17. For this purpose, we used 0.5 μM, 1 μM, and 2 μM human rCXCL17, concentrations at which this chemokine shows an important killing activity against other pathogens (3). Of note, human rCXCL17 showed no effect on *M. tuberculosis* HN878 growth at any concentration (Fig. 4A) compared with rifampicin, whereas only a modest reduction of the survival of *M. tuberculosis* H37Rv was observed at 2 μM (Fig. 4B). Hence, these findings indicate that CXCL17 lacks direct lytic capacity to kill *M. tuberculosis* bacilli.

## DISCUSSION

Several chemokine/chemokine receptor axes are essential for immune protection against *M. tuberculosis* (1). Some mediate beneficial activities, such as the adequate localization of different leukocyte subtypes at the site of *M. tuberculosis* infection. For instance, CXCL13 promotes the recruitment of CXCR5^+^ follicular B- and T helper cells into the *M. tuberculosis*–infected lung, where they form ectopic lymphoid follicles that support protective immunity against TB (14). Similarly, CCR2 signaling aids AMs in exiting airways and migrating to lung granulomas, and the absence of CCR2 leads to increased susceptibility to TB (7). Conversely, other chemotactic factors (i.e., S100A8/A9) mediate the accumulation of neutrophils into granulomas, exacerbating inflammation (15). Despite these advances, novel chemotactic factors of unknown function during TB have been recently discovered. This is the case for CXCL17, a novel mucosal chemokine constitutively produced along the respiratory tract (2, 3, 5), whose expression is further potentiated by inflammatory and microbial stimuli (4, 6). CXCL17 is a potential participant in the immunity of the lung. However, little literature exists about its implication during respiratory infections. Thus, our study is among the first to address the role of CXCL17 in immunity against a relevant respiratory pathogen such as *M. tuberculosis*.

CXCL17 mediates the recruitment of myeloid cells to the lung (4). Among cell subtypes responding to CXCL17 are DCs, monocytes, and macrophages (2, 4, 6). In this study, we confirmed these chemotactic properties of CXCL17, as we found that intratracheal administration of rCXCL17 to *M. tuberculosis* HN878-infected mice increased the accumulation of mDCs, monocytes, and RMs into the lung. Moreover, *M. tuberculosis*–infected CXCL17^−/−^ mice cannot recruit monocytes and RMs but have no deficiencies in lung mDCs. In this regard, it has been proposed that the activity of CXCL17 is only relevant to recruit DCs into uninflamed tissues (6). This may explain why CXCL17 was dispensable for chemotaxis of mDCs to the lung of *M. tuberculosis* HN878-infected CXCL17^−/−^ mice, whereas a higher number of mDCs accumulated into the lung of mice receiving exogenous rCXCL17 since early dpi, when the degree of inflammation was low. Alternatively, our findings suggest that, besides CXCL17, DCs may follow other redundant chemotactic signals to accumulate at the sites of *M. tuberculosis* exposure in mice (1).

Our data also show that a proportion of monocytes use the CXCL17 axis to infiltrate the lung during TB. Although the identity of the CXCL17R is yet controversial, monocytes expressing the G protein–coupled receptor 3 (GPR35), the proposed CXCL17R (16), might represent the myeloid population that migrated to the *M. tuberculosis*–infected lung in our experiments. GPR35 is constitutively expressed in inflammatory macrophages and downregulated upon LPS stimulation (17). Unfortunately, we did not address the expression of this receptor on monocytes/macrophages. Furthermore, little literature exists about a role for GPR35 during TB. However, our results also indicate that the subsets of monocytes responding to CXCL17 and their macrophage progeny may not exert relevant functions against *M. tuberculosis*, as their absence did not increase lung bacterial burden in CXCL17^−/−^ mice.

Other monocyte and macrophage subsets recruited by distinct chemokines may be more important for protective immunity against TB and compensate for the lack of CXCL17 activity in mice. Indeed, different myeloid cell subpopulations display a variable capacity to restrict *M. tuberculosis* growth (18). This functional variability depends on the origin of the phagocytes (18) and, perhaps, also on the chemokines mediating their recruitment, although the same chemokine axis can mediate the recruitment of permissive and resistant myeloid cell subsets. For instance, CCR2^+^ AMs mediate protective immunity against *M. tuberculosis* (7), whereas monocyte-derived macrophages responding to CCL-2 are more permissive and contribute to disease pathogenesis (19, 20). Collectively, these data suggest that the chemotactic capacity of CXCL17 is dispensable for control of *M. tuberculosis* HN878 infection in mice.

In contrast, our human data suggest a possible protective role for CXCL17 that is lost during PTB. This discrepancy might be related to a possible capacity of mice to make a compensatory response in the absence of CXCL17 through the production of other chemokines or cytokines that regulate migration or bactericidal activity of myeloid cells, as mentioned above. It is also probable that the levels of CXCL17 are downregulated in humans during PTB because of the increased activation of other chemokine axes. Conversely, some activities of CXCL17, such as its chemotactic functions, may be more important in humans than in mice. Thus, future studies should evaluate the capacity of myeloid cells from patients with different forms of TB to migrate in response to CXCL17.

Bactericidal activity of CXCL17 against respiratory microorganisms (3), including *M. tuberculosis*, might also differ between humans and mice. Interestingly, high expression of CXCL17 is observed at the luminal surface of the human alveolar epithelium, where it could act as an antimicrobial peptide (2, 3). To our knowledge, the bactericidal capacity of CXCL17 to lyse *M. tuberculosis* has not been tested before. Nonetheless, our results demonstrate that human CXCL17 lacks a bactericidal capacity to directly kill *M. tuberculosis* in vitro. Moreover, our findings in CXCL17^−/−^ mice also indicate that this activity is not relevant for *M. tuberculosis* clearance in vivo. Despite this, further analyses are required to evaluate whether CXCL17 potentiates the bactericidal activity of human and mouse macrophages or changes their activation status when challenged with *M. tuberculosis*. Importantly, the role of CXCL17 in TB immunity should be further addressed in murine models of infection with different *M. tuberculosis* strains and in larger cohorts of TB patients as well.

In summary, our data revealed that *M. tuberculosis* infection induces the production of CXCL17 in the mouse lungs in vivo and by epithelial and myeloid cells in vitro. In addition, CXCL17 recruits monocytes to sites of *M. tuberculosis* infection inside mouse lungs. In humans, lower serum CXCL17 levels are observed among active PTB patients when compared with subjects with LTBI, suggesting a protective role. However, the administration of exogenous CXCL17 to WT mice and the absence of CXCL17 signaling in knockout animals do not alter the susceptibility to TB in the murine model of hypervirulent *M. tuberculosis* HN878 infection. These findings suggest that, despite robust induction of CXCL17, this chemokine may have a redundant role during *M. tuberculosis* infection. Hence, our study expands the current knowledge of chemokine functions during TB.

## Abbreviations used in this article:

AM: alveolar macrophage
B6: C57BL/6
BMDC: bone marrow–derived DC
BMDM: bone marrow–derived macrophage
DC: dendritic cell
dpi: day post–*M. tuberculosis* HN878 infection
GPR35: G protein–coupled receptor 3
HC: healthy control
LTBI: latent TB-infected
mDC: myeloid dendritic cell
PTB: pulmonary TB
RM: recruited macrophage
TB: tuberculosis
WT: wild-type
